# The Pomegranate Deciduous Trait Is Genetically Controlled by a *PgPolyQ*-*MADS* Gene

**DOI:** 10.3389/fpls.2022.870207

**Published:** 2022-04-29

**Authors:** Rotem Harel-Beja, Ron Ophir, Amir Sherman, Ravit Eshed, Ada Rozen, Taly Trainin, Adi Doron-Faigenboim, Ofir Tal, Irit Bar-Yaakov, Doron Holland

**Affiliations:** ^1^Department of Fruit Tree Sciences, Institute of Plant Sciences, Agricultural Research Organization - The Volcani Center, Newe Ya’ar Research Center, Ramat Yishai, Israel; ^2^Department of Fruit Tree Sciences, Institute of Plant Sciences, Agricultural Research Organization - The Volcani Center, Rishon LeZion, Israel; ^3^Department of Vegetable and Field Crops, Institute of Plant Sciences, Agricultural Research Organization - The Volcani Center, Rishon LeZion, Israel; ^4^Institute of Plant Sciences, Newe Ya’ar Research Center, The Agricultural Research Organization - The Volcani Center, Ramat Yishai, Israel

**Keywords:** evergreen, genetic-map, poly-glutamine, thermo-sensor, dormancy, *Punica granatum*

## Abstract

The pomegranate (*Punica granatum* L.) is a deciduous fruit tree that grows worldwide. However, there are variants, which stay green in mild winter conditions and are determined evergreen. The evergreen trait is of commercial and scientific importance as it extends the period of fruit production and provides opportunity to identify genetic functions that are involved in sensing environmental cues. Several different evergreen pomegranate accessions from different genetic sources grow in the Israeli pomegranate collection. The leaves of deciduous pomegranates begin to lose chlorophyll during mid of September, while evergreen accessions continue to generate new buds. When winter temperature decreases 10°C, evergreen variants cease growing, but as soon as temperatures arise budding starts, weeks before the response of the deciduous varieties. In order to understand the genetic components that control the evergreen/deciduous phenotype, several segregating populations were constructed, and high-resolution genetic maps were assembled. Analysis of three segregating populations showed that the evergreen/deciduous trait in pomegranate is controlled by one major gene that mapped to linkage group 3. Fine mapping with advanced F3 and F4 populations and data from the pomegranate genome sequences revealed that a gene encoding for a putative and unique MADS transcription factor (*PgPolyQ-MADS*) is responsible for the evergreen trait. Ectopic expression of *PgPolyQ-MADS* in Arabidopsis generated small plants and early flowering. The deduced protein of *PgPolyQ-MADS* includes eight glutamines (polyQ) at the N-terminus. Three-dimensional protein model suggests that the polyQ domain structure might be involved in DNA binding of PgMADS. Interestingly, all the evergreen pomegranate varieties contain a mutation within the polyQ that cause a stop codon at the N terminal. The polyQ domain of PgPolyQ–MADS resembles that of the ELF3 prion-like domain recently reported to act as a thermo-sensor in Arabidopsis, suggesting that similar function could be attributed to PgPolyQ-MADS protein in control of dormancy. The study of the evergreen trait broadens our understanding of the molecular mechanism related to response to environmental cues. This enables the development of new cultivars that are better adapted to a wide range of climatic conditions.

## Introduction

Deciduous plants respond to decreasing temperatures and short day light by entering dormancy in winter (Penfield, 2008; Beauvieux et al., 2018; De Smet et al., 2021). This phenomenon occurs in many fruit tree species and is thoroughly studied due to its agricultural importance. The behavior of trees in changing climate and their ability to flower and bear fruit is dramatically influenced by temperatures during winter. Deciduous plants appear to be able to sense temperature and respond accordingly by entering or breaking dormancy (Luedeling, 2012). Several mathematical models were developed to quantify the amount of chilling required (CR) for plants to enter dormancy and to obtain optimal dormancy break (Richardson et al., 1974; Fishman et al., 1987; Dennis, 2003; Luedeling, 2012). The ability of plants to respond to chilling is controlled genetically (Rodriguez-A et al., 1994; Wang et al., 2002; Olukolu et al., 2009). It was shown that CR differ among different plant species and among cultivars of the same species (Olukolu et al., 2009; Trainin et al., 2013; Guo et al., 2014). A significant progress in understanding the genetics of CR in fruit trees was achieved when a peach evergreen mutant was mapped and the *dormancy associated MADS-box* genes (*DAM*) were discovered (Diaz, 1974; Wang et al., 2002; Bielenberg et al., 2008; Fan et al., 2010). It appears that the meristem of the evergreen peach mutant continues to be active during winter indifferently to chilling exposure (Diaz, 1974). Mapping chilling requirements of peach and apricot (Diaz, 1974; Fan et al., 2010) revealed a major quantitative trait locus (QTL), which corresponded to the location of the *DAM* genes. Over-expression of *Prunus DAM6* in apple plants caused inhibition of growth, repressed bud break competency of dormant buds, and a delay in bud outgrowth (Yamane et al., 2019). The *DAM* genes are a group of transcriptional factors that belong to the SVP/AGL24-type MADS-box proteins which are found in plants and animal cells and are involved in a large variety of activities including meristem identity and response to low temperatures (Shore and Sharrocks, 1995; Theissen et al., 2000). In addition to the *DAM* genes, other MADS box genes were found to be involved in modulation of the response to chilling (Hemming and Trevaskis, 2011). In Arabidopsis, the *MADS box SOC1* (*AGL20*) gene integrates vernalization and gibberellin signals for flowering signals (Moon et al., 2003). A *SOC1-like* gene was found to be associated with chilling requirements in apricot (Trainin et al., 2013). The SOC1 protein was shown to interact with other MADS box proteins in Arabidopsis (de Folter et al., 2005; Immink et al., 2012). Similarly, the SOC1 ortholog of Japanese apricot interacts with PmDAM6 (Kitamura et al., 2016). Over expression of the *SOC1 like* gene affects the duration of dormancy in Kiwi (Voogd et al., 2015) and promotes bud break in poplar (Gómez-Soto et al., 2021). Thus, genetic and functional analysis in deciduous fruit trees clearly suggest that MADS box genes could play a major role in mediating the response to chill and control of dormancy. Despite of the rapid progress in understanding the response of plants to chilling, the mechanism of action of MADS box genes in deciduous fruit trees is not yet clear.

Pomegranate is a deciduous fruit tree that grows in highly divergent geographical regions. It performs best in Mediterranean climate with relatively warm winter conditions (Holland et al., 2009). The Agricultural Research Organization (ARO) pomegranate collection in Israel is composed of representative species from diverse geographical regions of the world (Ophir et al., 2014), including five different species which display the evergreen phenotype (Nalawadi et al., 1973; Sharma and Dhillon, 2002; Holland et al., 2009; Borochov-Neori et al., 2011). Evergreen cultivars can grow and flower throughout the year in southern India mild winters (Teixeira da Silva et al., 2013). Similar behavior was observed for two evergreen pomegranate varieties in the desert climate of the Israeli southern Arava Valley (lat. 29°53′′N; 96 long. 35°3′′E) (Borochov-Neori et al., 2011). However, those cultivars shed their leaves in colder climates (Holland et al., 2009). This phenotype is comparable to the peach evergreen mutant where its axillary buds continue to develop despite exposure of the plants to low temperatures and short daylength (Diaz, 1974; Rodriguez-A et al., 1994). The ability of the evergreen pomegranate to generate flowers and bear fruit in warm winters is used by Indian growers to manipulate production and produce fruit throughout the entire year (Sharma and Dhillon, 2002). It appears that the origin of the evergreen pomegranate species is from India and central Asia as it was mainly found in these geographical locations (Sharma and Dhillon, 2002; Holland et al., 2009; Ophir et al., 2014).

Pomegranate is highly amenable to genetic research due to several characters. It is self-fertile, it produces a high number of hybrid seeds following a single hybridization event (Holland et al., 2009), and the tree bears fruit already at its second year. Additionally, it has a small (356.98 Mb) diploid genome (2*n* = 18) (Raman et al., 1971; Qin et al., 2017). These advantages allow generation of large segregating populations and a relatively rapid phenotype analysis. Development of DNA markers (Ophir et al., 2014) facilitated the establishment of a genetic map that enabled the identification of genes involved in color production (Trainin et al., 2021) and QTLs for fruit quality and plant height in pomegranate (Harel Beja et al., 2015). Another support to the pomegranate genetic research are three genomic sequences reported from two different cultivars (Qin et al., 2017; Yuan et al., 2017; Luo et al., 2020). In this study, we undertook a fine mapping approach using high throughput screening of segregating populations, and gene expression analysis to identify a novel gene associated with the evergreen/deciduous trait. We report here that this gene encodes for a unique polyQ-MADS domain protein. This study broadens our knowledge on the diversity of genetic components that control chilling response in plants and suggests a possible mechanistic explanation for some aspects of plant response to chill.

## Materials and Methods

### Plant Material

Three segregating F2 populations were planted and analyzed at the Newe Ya’ar Research Center in northern Israel: (1) ‘P.G.160-61 × P.G.100-1’ (*n* = 399) was constructed from a cross between ‘P.G.100-1’ (‘Wonderful’) a deciduous cultivar and (P.G.160-61) an evergreen cultivar originated from India. F1 plants, which were the progenitors of the F2 populations, were planted in 2006 and first flowers were self-pollinated at 2007. Five F2 populations were planted in 2008. (2) ‘Nana × Black’ (*n* = 295) was constructed from a cross between ‘Black’ (P.G.127–128) a deciduous cultivar and ‘Nana’ seedling selection (P.G.232-243, *Punica granatum* var. Nana) an evergreen non-deciduous cultivar. F1 plants, which were the progenitors of the F2 populations, were planted in 2008 and were self-pollinated at 2009. Four F2 population were planted in 2010. (3) ‘Nana × White’ (*n* = 267) was constructed from a cross between ‘White’ (P.G.164-65) a deciduous cultivar and evergreen ‘Nana’ seedling selection (P.G.232-243, *P. granatum* var. Nana). F1 plants, which were the progenitors of the F2 populations, were planted in 2010 and were self-pollinated at 2011. Three F2 populations were planted in 2012. The parents are part of the ARO collection that is located at the Newe Ya’ar Research Center^1^ (Holland et al., 2009) and were called according to the nomenclature as reported in Ophir et al. (2014).

Advanced F3 (*n* = 355) and F4 (*n* = 176) populations were developed for ‘P.G.160-61 × P.G.100-1.’ Five F2 and two F3 heterozygous plants were self-hybridized to develop F3 and F4 generations, respectively.

Additional evergreen varieties from the ARO’s pomegranate germplasm that were studied: P.G.145-46, P.G.149-50 (additional ‘Nana’ seedling selection) and P.G.227-238 (cultivar with sour fruits with “Black” peel).

### Phenotype Evaluation of Segregating Populations

A plant was described as having the evergreen habit when leaves did not turn yellow and shed during autumn-winter months (November–January). In addition, when flowering and budding started early, toward the end of winter (February).

The evergreen habit of the ‘P.G.160-61 × P.G.100-1’ F2 population was monitored over 3 years (2009–2012). The ‘Nana × Black’ F2 population was monitored over 5 years (2011–2016) and the ‘Nana × White’ F2 populations was monitored over 2 years (2014–2015). F3 and F4 populations were described through autumn and winter seasons after 2–5 years from planting.

**FIGURE 1:**
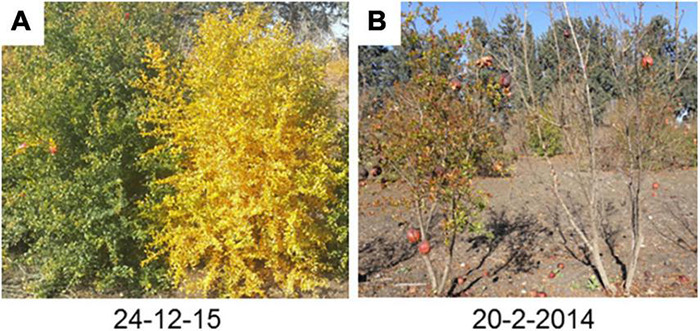
The evergreen and deciduous pomegranate behavior during autumn and winter. Evergreen and deciduous trees from segregating populations were photographed at December **(A)** and February **(B)**. The plant at the left **(A,B)** was an evergreen tree and at right was a deciduous tree.

In order to analyze the evergreen trait as a quantitative trait, dates when almost all leaves of the tree (estimated 60%) became yellow (Figure 1) and when budding started were recorded. The dates for ‘P.G.160-61 × P.G.100-1’ F2 population were indicated at 2011–2012 season and for ‘Nana × Black’ F2 population at 2011–2012 and 2012–2013 seasons. The dates were inverted to quantitative values by calculating days from November 1st to the recorded date. In addition, subtraction of the recording date when leaves became yellow from the date of budding gave the number of days that trees were without leaves.

### Chlorophyll Measurement

Chlorophyll content in the leaves was measured by chlorophyll content meter (CCM-200 plus, apogee, Logan, UT) along 21 months. Chlorophyll content is presented as chlorophyll concentration index (CCI) units. At least once a month, 35 representative leaves of two trees of the varieties P.G.100-1, P.G.160-61, and two F1 hybrids were measured.

### Chilling Effect on Budding

Cutting of P.G.145-46, P.G.160-61, P.G.100-1 were taken from trees in the orchard after the accumulation of 47 chilling hours (less than 7°C), to a warmed room (26°C) with 16 and 8 h of light and dark, respectively. Each cultivar had three replicates with ten stems of 30 cm length. Date of 50% budding was recorded. Budding capability was assessed as the number of days to 50% budding.

### DNA Extraction

The DNA-extraction protocol was based on Porebski et al. (1997) with few modifications as published in Ophir et al. (2014), young leaves (0.5 g) were resuspended in a 6 ml extraction buffer and the chloroform–octanol solution was replaced with chloroform–isoamyl alcohol. DNA was precipitated with sodium acetate instead of sodium chloride.

Crude DNA was extracted from young leaf (10 mg) in 96-well PCR plate according to a protocol based on Xin et al. (2003). DNA was diluted 1:100 for further analysis.

### Statistical Analysis

Means, standard deviations, trait distribution, pairwise correlation, ANOVA and Welch ANOVA, Shapiro–Wilk *W* Test, and Wilcoxon/Kruskal–Wallis Tests (Rank Sums) analyses were conducted with the JMP, v. 7.0 software (SAS Institute Inc., Cary, NC, United States).

## Genotyping

### Single Nucleotide Polymorphism Markers

#### Segregating Bulk Array Hybridization and Signal Preprocessing

Six segregating bulks were created from the F2 ‘P.G.160-61 × P.G.100-1’ populations originated from three different F1 plants, each population gave two groups, evergreen and deciduous (Supplementary Table 1). The evergreen group was formed from 22, 12, and 25 plants and the deciduous from 20, 14, and 34 plants, respectively. Each group was divided to two bulks to serve as biological replications. DNA was extracted from each bulk separately. Hybridization of evergreen bulk against deciduous bulk was conducted on two arrays replicates. Additionally, replicates of each of the bulks were hybridized as bulk against itself to measure within bulk variance. The between and within bulks’ variance was estimated using factorial design as described in Ophir et al. (2010). The probes of the SurePrint Agilent array were composed of custom self-designed based on 15,494 cDNA sequences as described in Harel Beja et al. (2015).

Signal preprocessing, scaling, and differential allele proportion assessment was analyzed using LIMMA R-package (Ritchie et al., 2015). Adjustment of false positive *p*-values that occur in multiple tests was calculated using Benjamini and Hochberg’s (1995) method.

#### Fluidigm Array Genotyping

Individual plants from one F2 population of ‘P.G.160-61 × P.G.100-1’ (*n* = 92) were genotyped with 201 SNPs (Supplementary Table 2), including 45 SNPs that had the highest scores from the bulk array hybridization, which were expected to be mapped close to the evergreen trait, three additional new SNPs and 153 selected SNPs of the pomegranate 1,092 mapped SNPs (Harel Beja et al., 2015). Those SNPs were selected since they were polymorphic between P.G.100-1 and P.G.160-61 and were dispersed along the ‘Nana × Black’ genetic map (Harel Beja et al., 2015). SNPs were developed by Fluidigm Corporation (South San Francisco, CA, United States). SNP assays were run on FR48.48 arrays using EP1 Fluidigm platform. Technical procedure is the same as described in the company manual.^2^ Fluidigm genotyping raw data were converted into table where rows are markers’ assays and columns are accessions’ samples. Genotype data of 181 SNPs was used to build the genetic map.

#### Single Nucleotide Polymorphism Genotyping by TaqMan and Sequencing

Plants from F3 and F4 populations (243 and 176, respectively) were screened with two markers (c26365 and c8339) by TaqMan Genotyping on StepOne Plus (Applied Biosystems, Foster City, CA, United States) as indicated in the Applied Biosystems StepOne™ protocol.

Additional SNP markers (Supplementary Table 3) were genotyped by sequencing of PCR products with the 3130 Genetic Analyzer (Applied Biosystems, Foster City, CA, United States).

### Simple-Sequence Repeats Markers

Simple-sequence repeats markers were developed from genomic pomegranate sequences (Supplementary Table 3). SSR amplification reaction was carried out in 20 μl volume containing template ∼30 ng gDNA, 2.0X Apex Taq RED master mix kit (Genesee Scientific, El Cajon, CA, United States) and 0.4 μM each primer (Supplementary Table 3). Thermal cycling conditions were: 5 min at 94°C, followed by 30 amplification cycles at 94°C for 15 s, 58°C for 30 s, and 72°C for 30 s and then a final extension at 72°C for 45 min. Genotyping was performed using GENESCAN-500-LIZ size standard (Applied Biosystems, Foster City, CA, United States) on a 3130 xl Genetic Analyzer (Applied Biosystems, Foster City, CA, United States) and analyzed using the GeneMapper software (Applied Biosystems, Foster City, CA, United States).

### Map Construction and QTL Analysis

Mapping was performed using JoinMap 3.0 software (Van Ooijen and Voorrips, 2001). Markers were grouped at a minimum LOD score of 4.0 and a recombination frequency value of 0.4. JoinMap 3.0 software uses Kosambi mapping functions to translate recombination frequency into map distance.

Using the MapQTL^®^ 5 software (Van Ooijen, 2004), QTLs and their significance were calculated using interval mapping (IM), multiple QTL model (MQM), and permutation analysis. A QTL was determined significant when its LOD score was higher than the calculated threshold (1000 permutation at *p* = 0.05).

### RNA Extraction

Two varieties, P.G.100-1 and P.G.160-60, the parents of the segregating populations, as well as eighteen representatives of the F3 population (nine evergreen and nine deciduous) were selected for RNAseq analysis. Selected F3 plants had a homozygous genotype at the c26365, highly associated marker to the evergreen trait. In addition, they were described as evergreen or deciduous plants during 2 years (2013–2015). Chlorophyll content was examined for P.G.100-1 and P.G.160-60 and the selected F3 plants at three dates: 24/8/2016, 25/9/2016, and 9/11/2016.

A sample consisted of leaves collected at 25/9/2016 from different branches on the same tree. Tissues immediately frozen in liquid nitrogen were crushed and kept at -80°C until further analysis. Total RNA was extracted as described in Trainin et al. (2021) for each plant individually. Samples for RNAseq analysis consisted of three and two biological replicates (taken from different trees) of P.G.100-1 and P.G.160-61, respectively, and three mixed RNA bulks of evergreen and deciduous seedlings, each hold three different plants. Construction and sequencing of mRNA-seq libraries were performed at the Technion Genome Center.^3^ Briefly, mRNAseq libraries were generated from total RNA, using the TruSeq RNA protocol. The indexed libraries were pooled and subjected to sequencing on an Illumina HiSeq2500 instrument (Illumina, San Diego, CA, United States), 50 bp single read.

### Transcriptome Analysis

Raw-reads were subjected to a filtering and cleaning procedure. First, raw reads were filtered and using Trimmomatic (Bolger et al., 2014) to remove adapters. Next, the FASTX Toolkit^4^ was used to trim read-end nucleotides with quality scores < 30, using the FASTQ Quality Trimmer, and to remove reads with less than 70% base pairs with a quality score ≤ 30 using the FASTQ Quality Filter. Data was submitted to SRA bioproject PRJNA802080. Clean reads were mapped to the reference genome of *P. granatum*^5^ using Cufflinks RNA-Seq workflow^6^ using Tophat2 software (Kim et al., 2013) (v. 2.1) with an average mapping rate of 96.3%. Gene abundance estimation was performed using Cufflinks (Trapnell et al., 2010), (v. 2.2) combined with gene annotations (GCA_002201585.1_ASM220158v1). Gene expression values were computed as FPKM (Fragments per kilo base per million mapped reads). Differential expression analysis was completed using the DESeq2 R package (Love et al., 2014). Genes that were more than twofold differentially expressed with false discovery-corrected statistical significance of at most 0.05 were considered differentially expressed (Benjamini and Hochberg, 1995).

The gene sequences were used as a query term for a search of the NCBI non-redundant (nr) protein database that was carried out with the DIAMOND program (Buchfink et al., 2014). The search results were imported into Blast2GO version 4.0 for gene ontology (GO) assignments.

Transcripts were analyzed by the Integrative Genomics Viewer (IGV) interactive tool to visual explore their structure.

### Sequence Analysis of Pomegranate *PgPolyQ-MADS* Gene

The *PgPolyQ-MADS* full length transcript sequence of pomegranate was cloned and sequenced from ripe arils of P.G.200-211, a deciduous cultivar with a light pink aril color originated from Spain (Harel-Beja et al., 2019). Primers used for the full-length transcribed gene were 5610_9 and 5610_6 (Supplementary Table 4). PCR products were sequenced with 3130 Genetic Analyzer (Applied Biosystems, Foster City, CA, United States). NCBI Blastp was used to find homologous proteins and conserved domains.^7^

### Expression of *PgPolyQ-MADS* in *Arabidopsis*

Pomegranate *PgPolyQ-MADS* was cloned into the expression vector PBI121 (GenBank entry AF485783) under the control of the 35S promoter by Bio Basic Inc. (El Cajon, ON, Canada) (Supplementary Figure 1), the plasmid was designated PBI121-5610.

*Agrobacterium tumefaciens* strain GV3101 containing PBI121-5610 and PBI121 (control) were used to transform Arabidopsis (Col-0) by the floral dip in filtration method (Clough and Bent, 1998). Selection of transformants was conducted on 0.8% agar containing Murashige and Skoog (MS) salts (2.2 g/l) and kanamycin (50 μg/ml). Kanamycin-resistant seedlings (five of PBI121-5610 transformant and two of PBI121) were then transferred into soil to set seed. Progeny from self-fertilized primary transformants were grown in pots for phenotypic analyses at 20°C, 12 h light. PBI121-5610 transformant plants were verified by PCR with the primers 5610-5 and 35S_out (Supplementary Table 4). Control plants, transformants with PBI121, were verified by PCR with primers uidA forward and uidA reverse (Supplementary Table 4). Thirty-seven transgenic with PBI121-5610 Arabidopsis lines were obtained and characterized. Seeds were acquired from twelve positive lines for further analysis. Experiments were continued and repeated with two transgenic lines (#22 and #31) that showed strong characteristic phenotypes. Flowering time was recorded when first bolt had open flowers.

### Protein Modeling

Structure model was constructed using the intensive mode of Phyre2 (Kelley et al., 2015). Visualization and analyses were done using PyMol^8^ (Schrödinger and DeLano, 2020).

## Results

### Chlorophyll Content of Pomegranate Leaves and Temperature Effect

**FIGURE 2:**
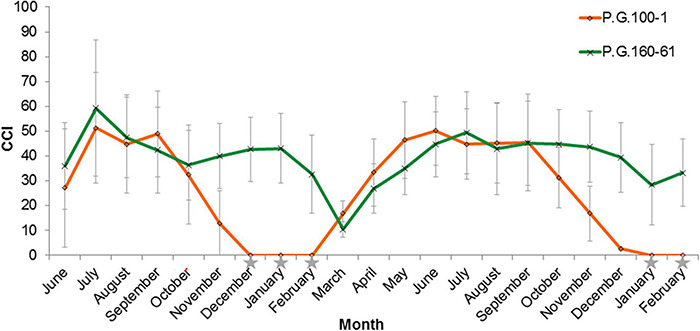
Chlorophyll content in the leaves of two pomegranate varieties and their hybrids along 21 months. Chlorophyll content index (CCI; 0–100) was determined along 21 months in the leaves of P.G.100-1 (Orange line, diamond signs), P.G.160-61 (Green line, x signs). The graph shows the averages CCI of 70 leaves with STDEV. Gray stars represent measurments’ dates when the average minimum temperature was below 10°C.

The leaves of deciduous pomegranate change their color in the autumn, while the evergreen varieties retain their green leaves and continue to generate new buds even during the winter (Figure 1). The latter phenomenon was titled evergreen. In order to quantify the differences between the different pomegranate varieties through time, chlorophyll content in the leaves was measured in two varieties along 21 months (June 2014–February 2016). Measurements are represented as chlorophyll concentration index (CCI). While the leaves of P.G.100-1 and those of the F1 hybrids lose their chlorophyll from September through February, the leaves of the evergreen variety, P.G.160-61, remain green during all year (Figure 2). However, when temperatures dropped below 10°C for few days or more, the leaves of the evergreen defined cultivar P.G.160-61 lost their chlorophyll due to cold injury and dropped. Four additional accessions (P.G.145-45, P.G.227-238, P.G.149-50, and P.G.160-61) in the ARO pomegranate collection showed the same behavior. This phenomenon led to determine pomegranate as a conditional deciduous plant.

The differential effect of chilling on the budding of evergreen and deciduous pomegranates was studied on stem cuttings. Cutting of evergreen varieties P.G.145-46 and P.G.160-61 and deciduous variety P.G.100-1 were cut from trees in the orchard after accumulating 47 chilling hours (less than 7°C), and then warmed to 26°C. Date of 50% budding was recorded. Budding capability was assessed as the number of days to 50% budding. The evergreen varieties showed significantly earlier budding capability (Tukey–Kramer test) than the deciduous variety. 50% of the branches of P.G.145-46 and P.G.160-61 had buds after 23 and 39 days, respectively, while 50% branches of P.G.100-1 had buds only after 58 days (Supplementary Table 4).

### Genetics of the Evergreen Trait in Pomegranate

In order to study the genetics of the evergreen trait, three different F2 populations, originated from five different parents (’P.G.160-61 × P.G.100-1,’ ‘Nana × Black,’ and ‘Nana × White’) were established. The distribution and segregation of the evergreen phenotype was analyzed in these populations through autumn and winter over 2 or 3 years (‘P.G.160-61 × P.G.100-1’ 2009–2012, ‘Nana × Black’ 2010–2012, and ‘Nana × White’ 2013–2015). Plants were described as evergreen when leaves remained green and did not drop during autumn-winter months (November–January), while flowering and budding started early, before spring (February). The evergreen phenotype was segregated in all the different F2 populations as a one major gene with significant (Chi square *p* > 0.05) 1:3 ratio (Table 1), where evergreen phenotype was recessive to the deciduous phenotype.

**TABLE 1:** The distribution of the evergreen and deciduous phenotypes in three F2 populations.

Population	Evergreen	Deciduous	Ratio of evergreen	Chi square *p* value
‘P.G.160-61 × P.G.100-1’	109	290	0.27	0.29
’Nana × Black’	79	216	0.27	0.48
’Nana × White’	67	200	0.25	0.97

*Probability of Chi square was calculated by http://stattrek.com/online-calculator/chi-square.aspx.*

### DNA Markers Associated With the Evergreen Trait

In order to find strongly linked markers to the evergreen trait, two divergent groups of plants were selected from the ‘P.G.160-61 × P.G.100-1’ F2 population. One group contained plants that expressed the evergreen phenotype and the second group expressed the deciduous phenotype. Comparative hybridization between pooled DNA from evergreen group and the deciduous group was performed on the SNP array, which was developed for pomegranate (Harel Beja et al., 2015). Differential signals were sorted from the strongest associated SNP to the least by sorting the differential signal ascending, yielding 133 statistically significant differential signals, which depicted SNPs (adjusted-*p* < 0.05), whereas 40 of them had greater confidence at (adjusted-*p* < 0.001) (Supplementary Table 6).

In addition, sequential SNP genotyping was performed using the Fluidigm technology with 201 SNPs for the ‘P.G.160-61 × P.G.100-1’ F2 population (*n* = 92). The markers used in this analysis included 45 SNPs with the highest scores (*p* < 0.0025) from the bulk array hybridization described above, three additional new SNPs and 153 SNPs from the pomegranate SNP subset (Harel Beja et al., 2015). Altogether, a total number of 181 SNPs were found appropriate for further analysis (Supplementary Table 2).

### Mapping the Evergreen Trait

A linkage map was built for the ‘P.G.160-61 × P.G.100-1’ F2 population (Supplementary Table 7) in addition to the published ‘Nana × Black’ map (Harel Beja et al., 2015). The new map contains 167 markers and is divided to 12 linkage groups (LGs). 130 markers were common with the published map and their order was identical except for a few mismatches: one in LG3, three in LG5 and three in LG8. LG6 of ‘P.G.160-61 × P.G.100-1’ map contained three markers that were mapped at LG10 and one at LG11 on the ‘Nana × Black’ map.

**FIGURE 3:**
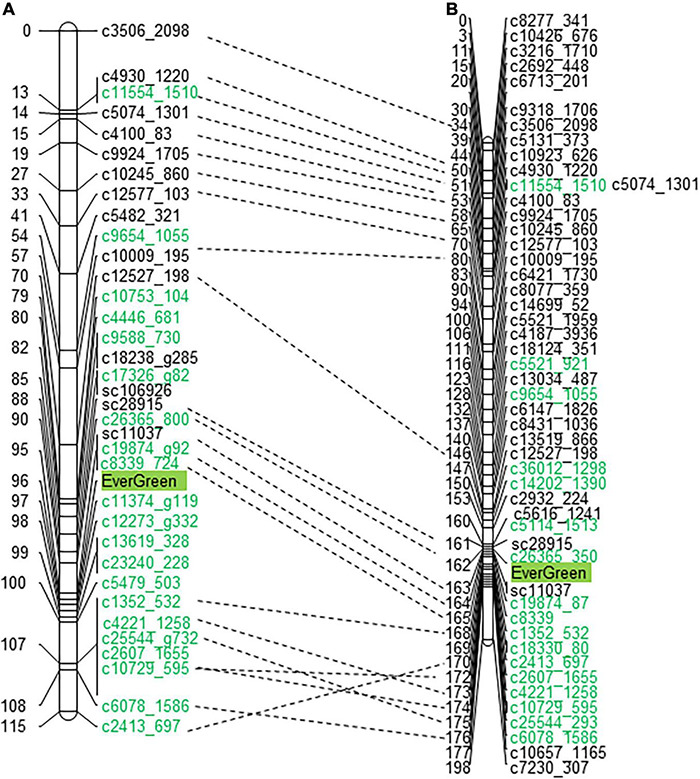
The evergreen trait was mapped to LG3 in two different F2 populations. The evergreen trait was mapped to LG3 of ‘P.G.160-61 × P.G.100-1’ **(A)** and ‘Nana × Black’ **(B)** genetic maps. Markers written in green were associated to the evergreen segregating bulks at the SNP CHIP. Dashed lines connect common markers.

The evergreen trait was mapped as a qualitative trait to LG3 at both linkage maps, at 98 and 163 cM (centimorgan) on ‘P.G.160-61 × P.G.100-1’ and ‘Nana × Black,’ respectively (Figure 3 and Supplementary Table 7). Four DNA markers were mapped at ‘P.G.160-61 × P.G.100-1’ map and two at the ‘Nana × Black’ map in the vicinity of the evergreen locus within approximately one cM.

A total of 70 and 38 of the bulk segregated associated SNP markers were mapped on ‘Nana × Black’ and ‘P.G.160-61 × P.G.100-1’ pomegranate linkage maps, respectively. They were mapped on 10 LGs at the ‘Nana × Black’ map, and at five LGs on the ‘P.G.160-61 × P.G.100-1’ map (Supplementary Table 7). The highly linked markers (*p* < 5^*E*–07^) were mapped to LG3 at the region where the evergreen trait was mapped (153–172 cM at the ‘Nana × Black’ and 79–107 at the ‘P.G.160-61 × P.G.100-1’ map).

### QTL Analysis to the Evergreen Trait

**FIGURE 4:**
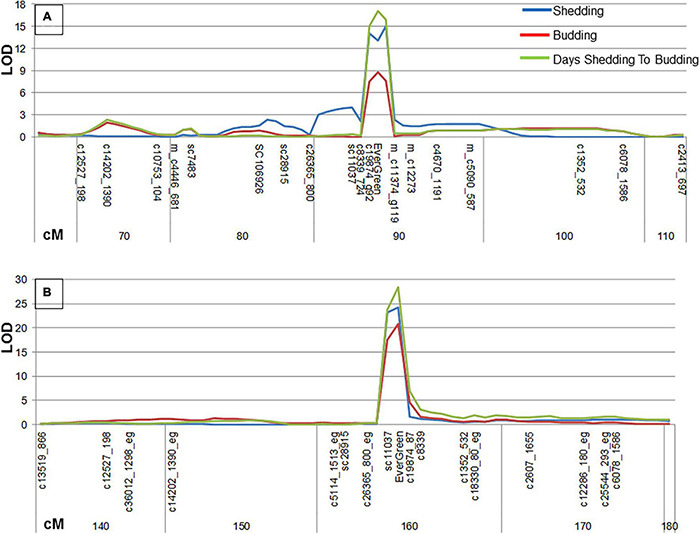
Three evergreen related traits were subjected to QTL analysis and mapped to LG3. MQM analysis for three traits: shedding (Blue, days from the date leaves were yellowing to November 1st), budding (Orange, days from the date of budding to November 1st), and days from shedding to budding (green). The analysis was done for ‘P.G.160-61 × P.G.100-1’ F2 population in 2011–2012 **(A)** and for ‘Nana × Black’ F2 population in 2012–1013 **(B)**. The x axis represents the markers as they ordered along the LG3 and cM distance.

The evergreen phenotype was divided to three traits: leaves shedding, budding and the time between leaves shedding and budding. The dates of each trait were indicated for ‘P.G.160-61 × P.G.100-1’ F2 population at 2011–2012 season and for ‘Nana × Black’ F2 population at 2011–2012 and 2012–2013 seasons. Each trait was quantified by calculating the number of days between November, 1st and the date the trait was recorded. All three traits were mapped, by MQM analysis, as one major QTL at LG3 for both F2 segregating populations (Figure 4) with 56–89% explanation of the trait by IM analysis, depending on the character measured, year and population (Supplementary Table 8). The highest LOD values (LOD > 7.4) were related to the EverGreen loci and to adjacent markers. Additional minor QTLs (LOD > 3.8) for the three traits were analyzed for ‘P.G.160-61 × P.G.100-1’ F2 population, by IM analysis, at LG7 (Supplementary Table 8).

### Positioning of the Evergreen Region in the Pomegranate Genome Sequence

**FIGURE 5:**
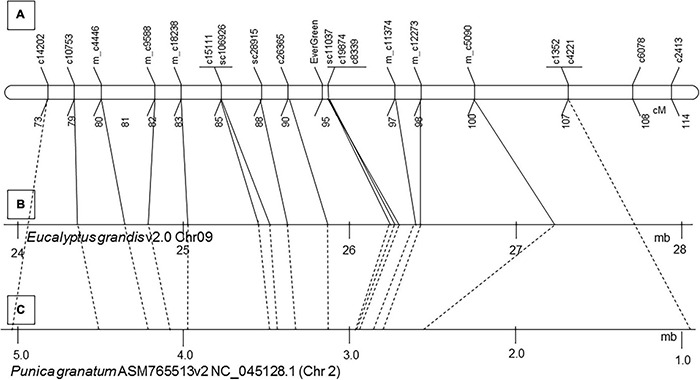
Microsynteny between pomegranate LG3, pomegranate ‘Tunisia’ genome sequence ASM765513v2 and the homologous genomic sequence of *Eucalyptus grandis* V1.0 chromosome 9. Twenty-seven pomegranate genes that were mapped in the vicinity of the evergreen trait at ‘P.G.160-61 × P.G.100-1’ map **(A)** were blasted to the *Eucalyptus grandis* V1.0 chromosome 9 **(B)** and to pomegranate ‘Tunisia’ genome ASM765513v2 **(C)**. Continuous lines connect the mapped pomegranate genes to their position on chromosome 9 of *Eucalyptus grandis* V1.0. Dashed lines connect the markers to their position on NC_045128.1 of pomegranate genomic sequence ASM765513v2.

Markers that were mapped close to the evergreen loci, 22 and 12 cM, above and below, respectively (Figure 5A), were blasted (BLASTN) to two pomegranate genome sequences: GCA_002201585.1 assembly of ‘Dabenzi’ pomegranate and ASM765513v2 of ‘Tunisia’ (Supplementary Table 9). The markers were ordered on MTKT01004399 of GCA_002201585.1 assembly and to NC_045128.1 scaffold (Chromosome 2) of ASM765513v2 according to their order on the genetic map (Figure 5C).

The 34 cM interval that included the “EverGreen” loci covered a region of 4,099,398 bp at NC_045128.1 (905,484–5,004,872 bp) scaffold, including 577 genes. Identification of the relevant genomic sequences facilitated the development of additional DNA markers that were used to genotype plants within advanced segregating populations (Supplementary Table 3).

### Microsynteny to Eucalyptus

Eucalyptus and pomegranate are members of the Myrtales order. It was of interest to study the synteny between the genomes at the evergreen region. Sequences of genes of the evergreen trait on the genetic map (‘P.G.160-61 × P.G.100-1’ 73 –99 cM), that comprised markers of around 25 cM were blasted (BLASTN) to the *Eucalyptus grandis* genome V1.0. Interestingly, the order of these gene markers on the pomegranate genetic map and on the pomegranate ‘Tunisia’ genome was in synteny to that of the genes at chromosome 9 of *E. grandis* (scaffold9:24.4-27.2 Mb) (Figure 5B and Supplementary Table 9).

The matching genomic pomegranate and Eucalyptus sequences enabled the development of additional markers, including six SSR and eight SNPs (Supplementary Table 3). Genotyping with those markers enriched the marker collection within the close region for additional accuracy of the genetic map by additional recombinations.

### Fine Mapping of the Evergreen Locus Through the Usage of Recombinants and Identification of the Gene Responsible for the Evergreen/Deciduous Phenotype

Mapping the two different F2 populations, ‘P.G.160-61 × P.G.100-1’ and ‘Nana × Black,’ suggested that the evergreen trait was located between two close markers at LG3: c26365 and c8339. In order to reduce the area by additional recombinations, advanced segregating populations were developed. Five F2 and two F3 plants from ‘P.G.160-61 × P.G.100-1,’ that were found as heterozygous at the closed markers of the trait, were selected for self-hybridization to develop additional F3 and F4 populations with 355 and 176 plants, respectively. All populations segregated for the evergreen trait.

The highly associated markers, c26365 and c8339, were selected for genotyping the advanced populations. Interestingly, there were no recombinants between c26365 and the trait, within the F2 ‘P.G.160-61 × P.G.100-1’ population, although they were mapped with a gap of 5 cM. However, c8339 was mapped only one cM apart from the trait to the other direction with two recombinations. Moreover, within the F2 ‘Nana × Black’ population there were two recombinants between c26365 and one between c8339 and the trait, respectively (Table 2). The physical distance between those markers, according to of pomegranate genome assembly ASM765513v2 is 176,366 bp.

**TABLE 2:** Recombinants between the evergreen trait and closed markers in four populations.

Population	’P.G.160-61 × 100-1’			‘Nana × Black’
Generation/Marker	F2	F3	F4	F2
c15111	7/99	nd	nd	nd
sc106926	5/102	1/5	4/10	nd
sc28915	3/102	1/21	3/10	3/76
sc174460	2/2	1/5	4/5	2/3
c26365	0/92	1/243	3/176	2/76
Pgr5601	nd	0/5	1/6	0/5
c31730	nd	0/5	1/9	0/3
195_146	nd	nd	1/1	nd
195_155	nd	nd	1/1	nd
5610	0/5	0/15	0/12	0/5
195_174	nd	2/2	1/1	nd
195_176	0/1	2/3	1/1	nd
195_183	0/1	2/3	1/1	nd
sc11037	1/102	1/21	4/11	0/76
c19874	2/190	nd	nd	0/76
c8339	2/92	3/243	4/176	1/76
sc280210	nd	3/4	nd	nd
sc7483	6/102	0/21	nd	nd

*The numbers describe how many recombinants were found out of the plants that were genotyped.*

*nd, not determined.*

Within the advanced populations, two plants out of 243 and three out of 176 were recombinants between c26365 marker and the evergreen trait among the F3 and F4, respectively (Table 2). Additional markers (Supplementary Table 3) were developed at the associated region according to corresponding Eucalyptus and pomegranate genomic sequences. Genotyping with close developed markers was performed for selected recombinant plants consistent with the recombination position (Table 2). Altogether, eighteen DNA markers were genotyped within the evergreen region (Supplementary Table 3). All the markers that were genotyped, exposed recombination with the trait, except for marker 5610. Therefore, it is suggested that 5610 is highly genetically associated to evergreen trait.

### MADS-Box Gene *AGL27* Is Genetically Responsible for Evergreen/Deciduous Trait

**FIGURE 6:**
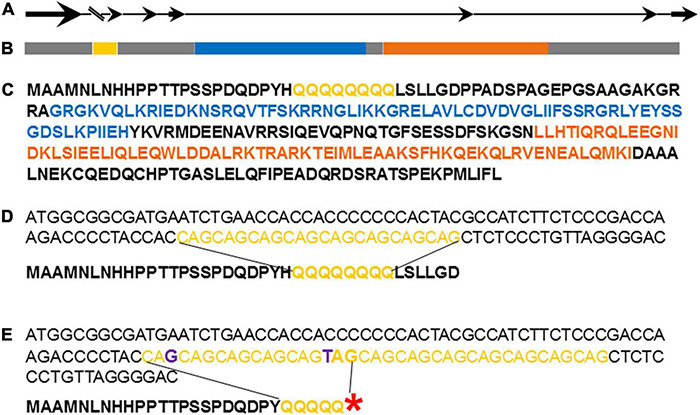
Graphic illustration of pomegranate PgPolyQ-MADS structure. Genomic composition of the gene **(A)**. Bold arrows represent axons, thin lines represent introns, and parallel lines represent first intron composed of ∼8,000 bp. LOC116195279 **(B)**, XP_031380211.1 isoform X1 protein sequence **(C)** and the polymorphism between P.G.100-1 **(D)** and P.G.160-61 **(E)** at the DNA and predicted amino acid sequences. Red asterisk represent stop codon caused by replacement of C by T. Yellow represents CAG repeats or poly glutamine, blue represents the MADS_MEF2_like conserved sequence, orange the K-box consensus sequence, and purple are the nucleotides changes between P.G.100-1 and P.G.160-61.

Closely linked markers’ sequences were blasted (BLASTN) to *P. granatum* isolate Tunisia-2019, ASM765513v2 whole genome shotgun sequence. Based on the recombinants at the advanced segregating populations the genomic region was reduced to 19,000 bp on chromosome 2 (NC_045128.1), between marker 195_155 and 195_174 (Supplementary Table 3). The marker 5610 was associated to the evergreen trait over all recombinant plants. The genomic associated region included only one gene and it is *LOC116195279* (XM_031524351.1; protein XP_031380211), which is annotated as agamous-like MADS-box protein AGL27. The gene is composed of seven exons that include two consensus sequences, a MADS_MEF2_like domain and a K-box consensus sequence (Figure 6). Interestingly, the gene contains an intron of almost 8,000 bp, which caused the separation of the gene into two genes (CDL15_Pgr005610 and CDL15_Pgr005611) at other pomegranate genome assembly (GCA_002201585.1 of ‘Dabenzi’ pomegranate). A repeat of eight successive glutamines (polyQ) is located at the N-terminal site of the deduced amino acid sequence, twenty-two amino acids subsequent the first methionine (Figure 6C). The gene was named *PgPolyQ-MADS*. The position of this small polyQ at the beginning of the protein could not be found in any other MADS-box proteins, by searching with BLASTP the available data bases.

Two polymorphic genetic changes were found between the deciduous P.G.100-1 and the evergreen P.G.160-61 at the *PgPolyQ-MADS* sequence. These changes are located within the CAG repeat located at the 5’ end of the gene (Figures 6D,E). The first change was that the evergreen P.G.160-61 had thirteen repeats, while P.G.100-1 had eight. Interestingly, the second change was at the sixth repeat of P.G.160-61, where a C was replaced by T, resulting in the formation of a stop codon (TAG) in the evergreen phenotype. P.G.160-61 and all other evergreen varieties present in the ARO pomegranate collection (P.G.145-46, P.G.149-50, P.G.227-238, and P.G.232-243) had the same genetic variation that caused a stop codon. Screening pomegranate accessions in the collection revealed that among the deciduous cultivars that were genotyped, twenty had identical allele to that of the *PgPolyQ-MADS* gene as P.G.100-1, one accession (P.G 221–232) had seven repeats and two seedlings from ‘Daru’ (P.G.263-274 and P.G.264-275) had nine repeats. However, none of the deciduous accessions showed the nucleotides replacements, which caused a stop codon (Supplementary Table 10).

### Three Dimensional Prediction Shows the polyQ Region Including the DNA Binding Domain Within the PgPolyQ-MADS Protein

**FIGURE 7:**
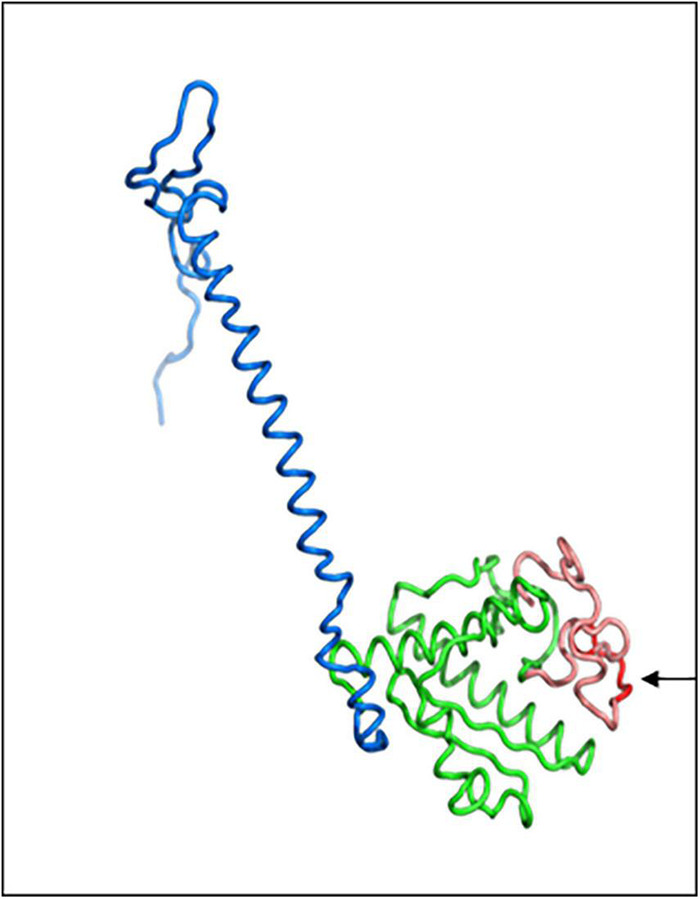
Three dimensional structure model of PgPolyQ-MADS transcription factor. Protein model was constructed by Phyre2. The model includes the N-terminal loop (salmon) including of eight glutamine residues (polyQ, red), DNA-binding domain (green), and the C-terminal keratin-like domain (blue). Arrow points to the polyQ residue.

In order to understand how the PgPolyQ-MADS box protein is influenced by the addition of the polyQ domain at the N terminal of the protein, the Phyere 2 program was used to model the protein (Figure 7). The monomeric protein is composed of two domains–DNA-binding domain and keratin like domain. The two domains were modeled with high confidence score, presenting up to 46% amino acid sequence identity to the templates used by Phyre2. The N-terminal polyQ tail and the C-terminal tail were predicted as disordered due to lack of available homologous structures for these areas. The model predicts that the polyQ domain is in close proximity to the DNA binding site and that its presence could influence its DNA binding capability. The polyQ domain might also play a role in targeting of other factors that could influence expression of the genes it binds to.

### Arabidopsis Transformants with PgPolyQ-MADS Had Early Flowering Phenotype

**FIGURE 8:**
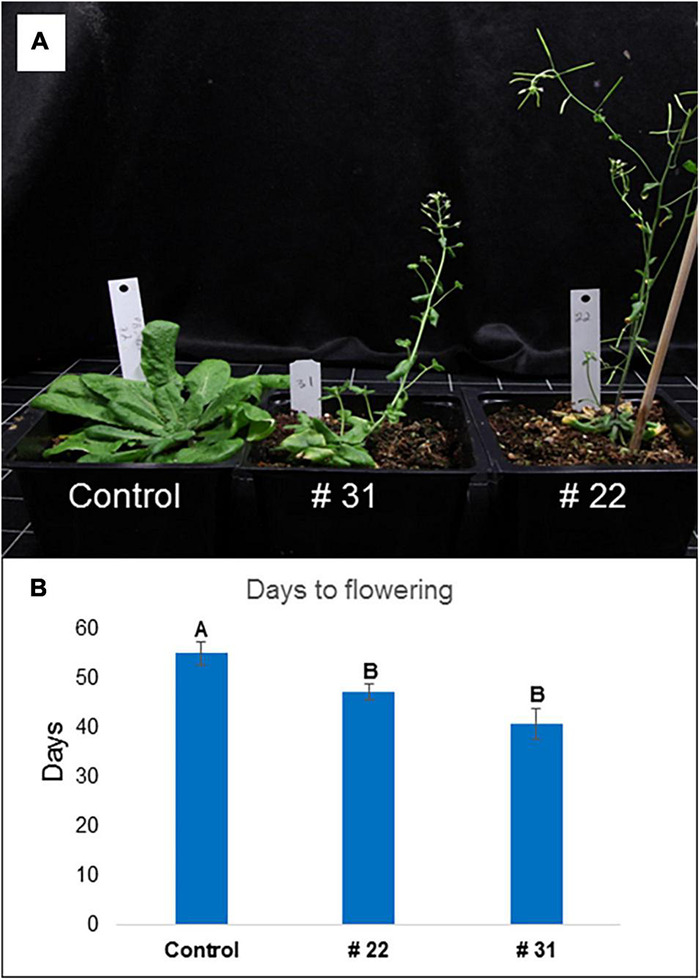
Arabidopsis transformants with *PgPolyQ-MADS* and control. Three representative transformant plants: Control plant transformed with PBI121, #31 and #22 plants transformed with *PgPolyQ-MADS*. **(A)** 45 days after sawing, #31 and #22 show a distinctive phenotype of smaller plants with few rolled leaves and early flowering. Days to flowering from sawing **(B)**, average ± standard error, letters represent significant differences by comparisons for each pair using Student’s *t* test.

Arabidopsis transformants (Col-0) containing the *PgPolyQ-MADS* gene originated from a deciduous cultivar (P.G.200-211) had a distinctive phenotype (Figure 8A). Effects included smaller plants, fewer, smaller and rolled leaves, and early flowering. Twenty-five transformed plants showed at least one of the effects and twelve had no effect. Days to flowering were reduced significantly (*p* < 0.01) from 52 ± 30.3 days at the control plants (T1, *n* = 8) to 37 ± 1.43 for the PgPolyQ-MADS transformants (T1, *n* = 36, one plant did not flower) (Supplementary Table 11). Seedlings of two transformed lines with the *PgPolyQ-MADS* gene that had a distinguished phenotype were grown for two additional experiments. Interestingly, those lines were originated from two different T0 plants. The extreme phenotype repeated (Figure 8A) and the number of days to flowering was reduced significantly (Figure 8B).

### Differential Transcriptome Between Deciduous and Evergreen Leaves

**FIGURE 9:**
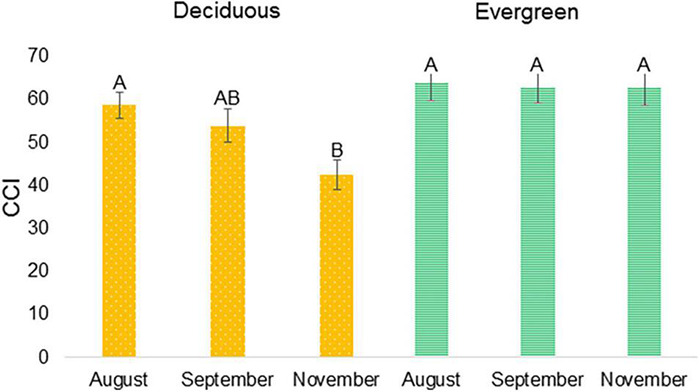
Chlorophyll content index (CCI) of deciduous and evergreen pomegranates at three dates. CCI was measured at 24.8.2016, 25.9.2016, and 9.11.2016 for deciduous (Orange, dots) P.G.100-1 and nine deciduous F3 descendants of ‘P.G.160-61 × Wonderful’ population and for evergreen (Green, stripes) P.G.160-61 and nine evergreen F3 progeny. Letters represent significantly differences by comparisons for each pair using Student’s *t* test.

Tracking chlorophyll content in the leaves along the year (Figure 2) showed that at the end of September deciduous varieties started to lose chlorophyll, while evergreen variety did not. That period was selected to study the different transcriptomes of deciduous and evergreen plants. P.G.100-1 and deciduous F3 progenies’ CCI values at 25.9.2016 (53.64 ± 12.52) were in-between the values at 24.8.2016 and 9.11.2016 (Figure 9). However, evergreen progenies and P.G.160-61 had mean CCI values of 67.76 ± 1.60 and were constant for all dates.

Transcriptome analysis was conducted for RNA extractions of leaves collected on 25.9.2016 from three P.G.100-1 trees and two P.G.160-61 trees. In addition, six bulks of F3 progenies from the ‘P.G.160-61 × Wonderful’ population were analyzed. Each bulk combined RNA from three different plants. Three bulks were from deciduous plants and three from evergreen plants. An average of 17,730,933 reads was obtained for each sample (Supplementary Table 12), with no considerable differences. Reads were mapped to pomegranate genome (GCA_002201585.1) where 32,582 contigs were analyzed.

Differential expression analysis showed forty-one transcripts that were differentially (*p* < 0.01) expressed between P.G.100-1 and P.G.160-61. These transcripts were also differentially expressed within the bulks of deciduous and bulks of the evergreen plants (Supplementary Table 13). Interestingly, these different genes included three genes from scaffold MTKT01004399.1 (GCA_002201585.1), which is associated to the evergreen trait. Those genes were annotated as acyltransferase and kinesin light chain. However, their location on the scaffold was not at the same loci as the associated markers were. Differential genes contain five transcription factors, including *MOTHER of FT and TF 1* (CDL15_Pgr019923, LOC116212806) which is located at MTKT01006319.1 (GCA_002201585.1).

Interestingly, transcript CDL15_Pgr005611 deduced to encode for the PgPolyQ-MADS was not differentially expressed between deciduous and evergreen plants, although the transcript in the evergreen plants contained the additional nucleotide assumed to cause for a stop codon. Thus, the evergreen alteration does not seem to change the transcription level of the gene. Transcription levels for transcript CDL15_Pgr005611 were with an average of 51.6 and 66.7 FPKM (Fragments per kilo base per million mapped reads) in P.G.100-1 and the deciduous bulks, respectively. The expression of the evergreen plants was 44.6 and 35.3 for P.G.160-61 and evergreen bulks, respectively.

## Discussion

Deciduous perennials as peach and plum include rare evergreen varieties (Diaz, 1974; Rodriguez-A et al., 1994). In pomegranate, this phenomenon is known to science and agricultural practice for many years (Diaz, 1974; Sharma and Dhillon, 2002; Holland et al., 2009). Indian growers use this trait to manipulate the time of pomegranate harvest to obtain yield throughout the year, which provides an economical gain. However, by manipulating the time of harvest, it is possible to divert the production period to seasons with more favorable environmental conditions that influence fruit quality, yield, irrigation and pest management. From a scientific point of view, the evergreen trait is of particular importance since it allows undertaking a genetic approach to find genes that are important for sensing the environment. Genetic studies of the evergreen peach mutant led to the identification of *DAM* genes in peach and several other fruit tree species that belong to the Rosacea family including, cherry (Castède et al., 2015), plum (Quesada-Traver et al., 2020), apple (Mimida et al., 2015), pear (Saito et al., 2015), and apricot (Olukolu et al., 2009; Trainin et al., 2013). However, despite the spike of accumulated data and knowledge, the sensing mechanism of environmental cues and the way in which plants translate this information to physiological processes is mostly unknown. This study exploited the existence of several different evergreen pomegranate accessions in the Israeli genetic collection to broaden the genetic knowledge on the evergreen trait and to shed light on the mechanism of action of the genes involved. Since pomegranate belongs to the Punicacea family which is evolutionary distant from the Rosacea family, this study may obtain broader genetic information that will uncover additional important aspects of dormancy. The physiological and genetic data gathered in our study on the pomegranate evergreen trait showed that a single recessive gene mainly controls it. This is true for the evergreen accessions, Nana and P.G.160-61 in our collection (Figures 3, 4 and Table 1). The evergreen tree constantly grow along the year in contrast to the deciduous tree that enters dormancy. However, exposure to low temperatures, below 10°C damage the leaves (Figure 2). Bud break will re-occur very fast once the temperatures increase. Thus, it appears that the same gene or QTL might control both entrance to dormancy and new bud development after cold damage. Indeed, three mapped evergreen related traits resulted as one major QTL at LG3. Two different evergreen pomegranate sources (P.G.160-61 and ‘Nana’) were mapped to that same region and are controlled by the same gene, entitled *PgPolyQ-MADS*. Former study suggested that the evergreen pomegranate accessions belong to a larger group of accessions, including deciduous accessions that originate from India and central Asia (Ophir et al., 2014). Because the same gene was discovered in the two different evergreen pomegranate accessions, it appears that the genetic event that formed the evergreen trait preceded the formation of the evergreen species.

The pomegranate and the peach evergreen trait have similar characteristics. In both species the trait is controlled by a main single recessive gene. Like in pomegranate, the peach meristem continues to grow in winter but the leaves suffer from cold damage and will eventually drop. The similarities between the peach and the pomegranate evergreen traits raised the question whether the genes that control them are similar. The mapping data indicate that the gene responsible for the evergreen trait in pomegranate is very similar to the Arabidopsis *AGL27 MADS box* gene (AT1G77080). The *DAM* genes associated to the trait in peach also belong to the MADS box genes but are more similar to the *AGL24* group (Bielenberg et al., 2008; Trainin et al., 2013). Like in *AGL27* from Arabidopsis the first two exons of *PgPolyQ-MADS* are separated by a large intron (Figure 6). Large introns were detected also in *SOC1* genes of apricot and peach (Trainin et al., 2013). In leafy spurge an alternative splicing was observed at the first exon, where the short transcript was upregulated only during endodormancy and the larger transcript was expressed during late endodormancy and throughout eco-dormancy (Horvath et al., 2017). Several studies on Arabidopsis suggest that it is a probable transcription factor involved in the negative regulation of flowering time in both long and short days, possibly through the photoperiodic and vernalization pathways and prevents premature flowering (Ratcliffe et al., 2001; Scortecci et al., 2003; Werner et al., 2005).

What is the function of *PgPolyQ-MADS* gene? The gene has a unique feature of additional domain which contains a repeat of eight glutamines (polyQ) and an additional 23 amino acids at the beginning of the deduced protein (Figure 6). However, the evergreen accessions possess a stop codon, caused by a change of C to T in the polyQ sequence, which eliminates the presence of the entire protein in the evergreen accessions (Figure 6). Protein modeling and three dimensional simulations [based on similar proteins with determined three dimensional structure (Theissen et al., 2000; Puranik et al., 2014)], suggest that the polyQ domain is positioned in close proximity to the DNA binding domain (Figure 7). Thus, the polyQ domain might function as a modulator of the DNA binding domain. Such a model was proposed for the action of a different transcriptional factor, the EARLY FLOWERING 3 (ELF3) DNA binding protein, which is involved in control of the circadian rhythm and control of flowering of Arabidopsis (Undurraga et al., 2012). ELF 3 contains a poly glutamine domain described as prion-like domain within the protein (Jung et al., 2020). It was suggested that the prion-like domain functions as a thermo-sensor. Remarkably, both proteins, the pomegranate PgPolyQ-MADS and the ELF3 from Arabidopsis function in mediating response to environmental cues, suggesting that the pomegranate protein PgPolyQ-MADS activity is mediating environmental cues through the polyQ domain. No other MADS box proteins with an N terminal polyQ domain were found in the genomes of other plants. However, another MADS domain protein, AGL3 (AT2G03710.1) with a poly glutamine at the C terminal was found in Arabidopsis (Huang et al., 1995). Interestingly, the PLAAC website (Lancaster et al., 2014) predicted that both MADS box proteins include the prion structure. In addition, variation in the polyQ repeat was detected in FCA protein among genus like *Triticum*, *Lolium*, *Oryza*, *Hordeum*, *Arabidopsis*, *Brassica*, *Pisum*, and *Medicago* (Lindqvist et al., 2007). FCA is a RNA-binding protein which is an abscisic acid receptor that was reported to be involved in thermosensing of flowering (Razem et al., 2006). It is to note that overexpression of PgPolyQ-MADS in transgenic Arabidopsis dramatically shortens the time to flowering (Figure 8) as was shown for Arabidopsis *AGL28* (Yoo et al., 2006) and for *Lilium formosanum MADS-box* gene (Liao et al., 2018). Pomegranate ELF3 (XP_031371603.1) does not contain the poly glutamine domain, neither the pomegranate agamous-like MADS-box protein MADS2 (XP_031407624.1), which was found to be most similar to AGL3, does not include the poly glutamine region. Thus, the presence and particular function of the PgPolyQ-MADS protein in pomegranate seems to be unique to pomegranate. Yet, the utilization of the polyQ domain could be a general mechanism in plants in controlling temperature response. The pomegranate FCA protein (XP_031405132.1) includes the poly glutamine motive as in Arabidopsis. The pomegranate data demonstrate that proteins, other than, ELF3 control temperature sensitive responses by using similar polyQ domains, as does the polyQ-MADS domain hybrid protein in conferring the evergreen phenotype.

Transcriptomic analysis did not show major gene expression differences between deciduous and evergreen varieties at the physiological stage of leaves that was analyzed. Only forty-one genes were found that show differential expression at confidence of less than 0.001. These include five transcriptional factors (Supplementary Table 13). One of them (Mother of FT) is associated with control of seed germination *via* the ABA growth regulator (Xi et al., 2010). In addition, the homologous gene to Mother of FT, the *FT* gene in popular, was shown to be involved in the control of flowering, growth cessation induced by short day and the bud set at fall (Böhlenius et al., 2006). Poplar has two *FT* paralogs, only *FT1* was shown to be involved in regulation of vegetative bud dormancy (Busov, 2019). Interestingly, dormancy inducing conditions in leafy spurge caused an opposite expression pattern of *DAM* and *FT* genes. During endodormancy induction *DAM* genes expression increased, while *FT* expression was suppressed (Hao et al., 2015). No significant differences in the level of expression of the *PgPolyQ-MADS* gene were observed, supporting the assumption that the evergreen trait is expressed due to the absence of a functional PgPolyQ-MADS protein in evergreen accessions. Our data combined with the data from ELF3 function strongly suggest the existence of a new control mechanism for sensing environmental signals through the control of transcriptional factor DNA binding. We propose that a poly glutamine domain could play an important role in this mechanism. The *PgPolyQ-MADS* could be a major temperature-sensing element in pomegranate that senses temperature through DNA binding and controls leaf shed and dormancy break through its activity as a transcriptional factor.

## Data Availability Statement

The datasets presented in this study can be found in online repositories. The names of the repository/repositories and accession number(s) can be found below: National Center for Biotechnology Information (NCBI) BioProject database under accession number PRJNA802080. The BioSample accessions: SAMN25511762, SAMN25511763, SAMN25511768, and SAMN25511769 for P.G.100-1, P.G.160-60, deciduous F3, and evergreen F3, respectively.

## Author Contributions

RH-B performed the experiments, analyzed the study, and wrote the manuscript. AS, RO, and AD-F contributed to bioinformatics. RE, AR, TT, and IB-Y performed the experiment. OT did the protein structure analysis. DH designed and conducted the research. All authors contributed to the article and approved the submitted version.

## Conflict of Interest

The authors declare that the research was conducted in the absence of any commercial or financial relationships that could be construed as a potential conflict of interest.

## Publisher’s Note

All claims expressed in this article are solely those of the authors and do not necessarily represent those of their affiliated organizations, or those of the publisher, the editors and the reviewers. Any product that may be evaluated in this article, or claim that may be made by its manufacturer, is not guaranteed or endorsed by the publisher.

## Footnotes

1
https://igb.agri.gov.il/web/index.php

2
www.fluidigm.com

3
https://tgc.net.technion.ac.il

4 http://hannonlab.cshl.edu/fastx_toolkit/index.html, version 0.0.13.2

5
https://www.ncbi.nlm.nih.gov/assembly/GCA_002201585.1

6
http://cole-trapnell-lab.github.io/cufflinks/manual/

7
https://blast.ncbi.nlm.nih.gov/Blast.cgi

8
https://www.pymol.org/pymol

## Abbreviations

ARO: Agricultural Research Organization
CCI: chlorophyll concentration index
cM: centimorgan
CR: chilling required
DAM: dormancy associated genes MADS-box
GO: gene ontology
IM: interval mapping
LG3: linkage group 3
MQM: multiple QTL model
nr: non-redundant
SNP: single nucleotide polymorphism
SSR: simple-sequence repeats
QTL: Quantitative Trait Locus.
