# Supplementary material for: The Pomegranate Deciduous Trait Is Genetically Controlled by a PgPolyQ-MADS Gene
Source: Front Plant Sci. 2022 Apr 29;13:870207. doi: 10.3389/fpls.2022.870207 (PMC9100744; doi:10.3389/fpls.2022.870207)
Supplement: Supplementary file 2 [file Image_1.pdf]

Supplementary Figures

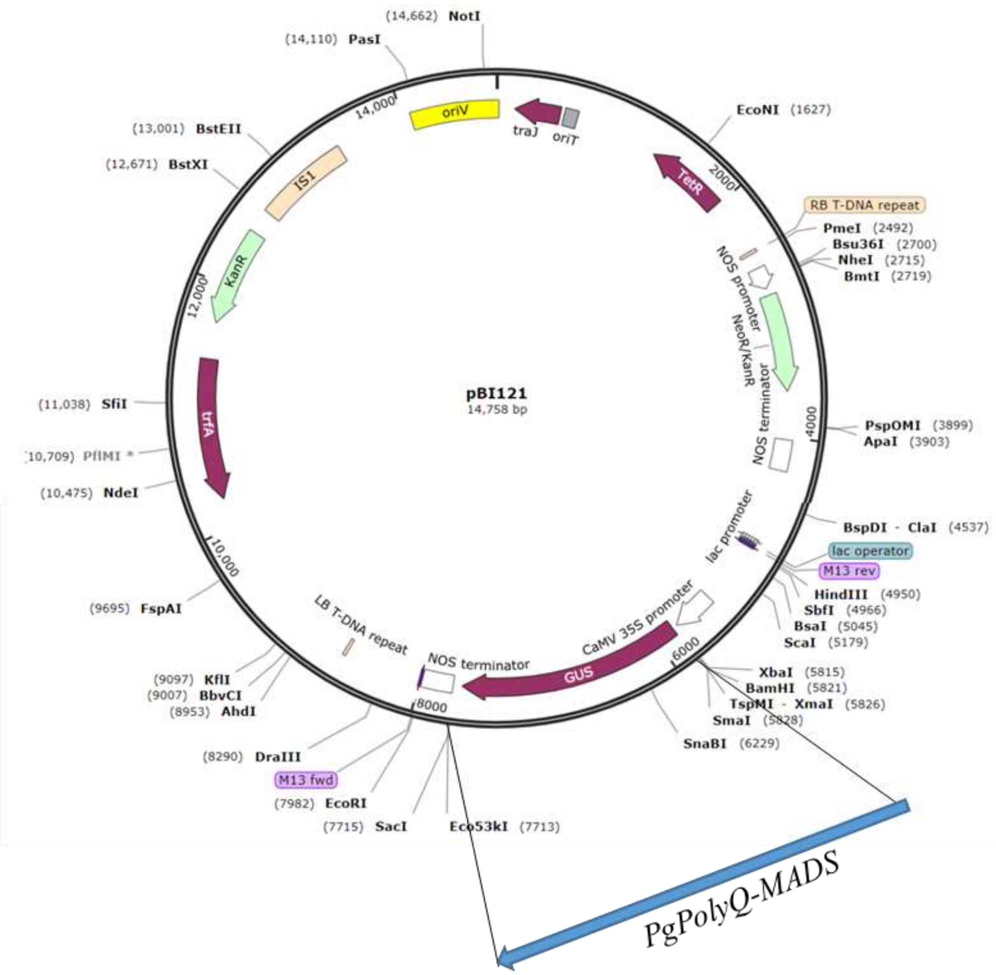

Supplementary Figure 1. The vector PBI121-5610 express the *PgPolyQ-MADS* gene. The *PgPolyQ-MADS* gene was cloned into the expression vector PBI121 (GenBank entry AF485783) under the control of the 35S promoter, instead of GUS. The plasmid was designated PBI121-5610. PBI121 vector graphic was downloaded from https://www.snapgene.com/resources/plasmid-files/?set=plant_vectors&plasmid=pBI121 website.
